# The effect of tongue elevation muscle training in patients with obstructive sleep apnea: A randomised controlled trial

**DOI:** 10.1111/joor.13369

**Published:** 2022-09-21

**Authors:** William Poncin, Nils Correvon, Jonathan Tam, Jean‐Christian Borel, Mathieu Berger, Giuseppe Liistro, Benny Mwenge, Raphael Heinzer, Olivier Contal

**Affiliations:** ^1^ School of Health Sciences (HESAV) HES‐SO University of Applied Sciences and Arts Western Switzerland Lausanne Switzerland; ^2^ Institute of Experimental and Clinical Research (IREC), pôle de Pneumologie, ORL et Dermatologie Université Catholique de Louvain Brussels Belgium; ^3^ Service de Pneumologie, Cliniques universitaires Saint‐Luc Brussels Belgium; ^4^ HES‐SO University of Applied Sciences and Arts Western Switzerland Lausanne Switzerland; ^5^ Service de Physiothérapie cardio‐respiratoire, département de chirurgie, cœur‐vaisseau et centre interdisciplinaire Centre Hospitalier Universitaire Vaudois Lausanne Switzerland; ^6^ AGIR à dom Meylan France; ^7^ Center for Investigation and Research in Sleep Centre Hospitalier Universitaire Vaudois and Université de Lausanne Lausanne Switzerland

**Keywords:** Iowa oral performance instrument, obstructive sleep apnoea, oropharyngeal Myofunctional therapy, Polygraphy, tongue muscle training, upper airway muscles

## Abstract

**Background:**

Oropharyngeal myofunctional therapy is a multi‐component therapy effective to reduce the severity of obstructive sleep apnoea (OSA). However, existing protocols are difficult to replicate in the clinical setting. There is a need to isolate the specific effectiveness of each component of the therapy.

**Objective:**

To assess the effects of a 6 weeks tongue elevation training programme in patients with OSA.

**Methods:**

We conducted a multicentre randomised controlled trial. Eligible participants were adults diagnosed with moderate OSA who presented low adherence to continuous positive airway pressure therapy (mean use <4 h per night). The intervention group completed a 6 weeks tongue elevation training protocol that consisted in anterior tongue elevation strength and endurance tasks with the Iowa Oral Performance Instrument. The control group completed a 6 weeks sham training protocol that involved expiratory muscle training at very low intensity. Polygraphy data, tongue force and endurance, and OSA symptoms were evaluated pre‐ and post‐intervention. The primary outcome was apneoa‐hypopnea index (AHI).

**Results:**

Twenty‐seven patients (55 ± 11 years) were recruited. According to modified intention‐to‐treat analysis (*n* = 25), changes in AHI and c did not significantly differ between groups. Daytime sleepiness (Epworth Sleepiness Scale) and tongue endurance significantly improved in the intervention group compared to the control group (*p* = .015 and .022, respectively). In the intervention group, 75% of participants had a decrease in daytime sleepiness that exceeded the minimal clinically important difference.

**Conclusion:**

Six weeks of tongue elevation muscle training had no effect on OSA severity.

## Graphical abstract

There is a need to elucidate the specific effect of a well‐defined component of orophareyngeal myofunctional therapy for patients with obstructive sleep apnea: tongue elevation muscle training. A 6‐week tongue elevation training programme did not change AHI or other polygraphy‐derived parameters compared to the control group. However, daytime sleepiness and tongue endurance significantly improved in the intervention group compared to the control group.

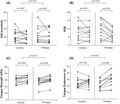

## 1 BACKGROUND

Obstructive sleep apnoea (OSA) is a common sleep disorder with an estimated prevalence of 19% in the general population of Lausanne (Switzerland).^1^ Untreated, OSA is an important cause of morbidity and mortality.^2^ Continuous positive airway pressure (CPAP) is the first‐line treatment option offered to these patients.^3^ However, between 17% and 85% of patients with OSA do not comply with CPAP therapy and remain untreated.^4^ Alternative treatment options have therefore been investigated, including myofunctional therapy (MFT) of the upper airways.^5^

The rationale for MFT stems from evidence showing that upper airway dilator muscles are dysfunctional and play a role in the pathophysiology of OSA.^6^ Indeed, this disorder is characterised by recurrent upper airway collapse that lead to partial or complete airway occlusion. Upper airway dilator muscles, including genioglossus which forms the bulk of the tongue and is the largest upper airway dilator, are pivotal in the maintenance of upper airway patency during sleep.^7^ However, poor genioglossus muscle activation during sleep is a physiologic feature contributing to OSA which, in susceptible individuals, may lead to the narrowing or collapse of upper airways.^8^ Moreover, histological changes in the genioglossus and reduced cerebrovascular reactivity in the motor areas that control the upper airway musculature are found in patients with OSA.^9^, ^10^ These neuromuscular deficits are associated with sensorimotor defects of the tongue^8^, ^11^ and result in a greater propensity to upper airway muscle fatigue.^9^

The MFT is a multi‐component intervention that typically comprises a combination of exercises covering various oropharyngeal structures, such as tongue, palate, pharynx, or epiglottis,^12^ and aims to act positively on the impaired sensorimotor deficits in the upper airway muscles encountered in patients with OSA.^9^, ^11^, ^13^, ^14^, ^15^ The efficacy of MFT in reducing the severity of OSA, daytime sleepiness and quality of life has been demonstrated in several reviews and meta‐analyses.^5^, ^16^, ^17^, ^18^, ^19^ Two previous randomised controlled trials conducted in patients with moderate OSA showed that, compared to sham therapy, MFT including soft palate, tongue, facial muscle exercises, as well as stomatognathic function exercises (i.e. suction, breathing, speech, swallowing, chewing) resulted in a reduction of apnoea‐hypopnea index (AHI) by 50% of its initial value.^12^, ^20^ The improvement in AHI seems related to upper airway remodelling, thereby reducing the collapsibility of upper airways during sleep.

However, since MFT protocols involve a variety of exercises,^20^, ^21^ the specific effectiveness of each exercise is unknown. Besides, the frequency and duration of existing protocols are challenging to replicate in the clinical setting.^20^ In addition, the time‐consuming aspect of this therapy might alter treatment adherence on the long run, which is considered as the main drawback of MFT.^22^ Identifying the effective parts of MFT and deciphering the underlying mechanisms of this multi‐component therapy may lead to a better understanding of how the therapy works and facilitate its implementation in the clinical setting. Assessing the efficacy of few, simple and easy exercises is thus needed. Because the genioglossus is the main upper airway dilator muscle,^23^ focusing on the tongue may represent an interesting anatomical target. Another argument that endorse the strategy to focus on the tongue is the presence of lower lingual tone in children and adults with sleep‐disordered breathing compared to healthy controls.^24^, ^25^, ^26^ Interestingly, recent studies have shown that the improvement of AHI with MFT correlates significantly with the improvement of tongue strength.^14^, ^27^, ^28^

Therefore, we aimed to assess the specific effectiveness of a tongue elevation muscle protocol in reducing OSA severity. We hypothesised that a 6 weeks tongue strength and endurance training programme would reduce AHI in patients with OSA.

## 2 METHODS

### 2.1 Design and participants

This is a multicentre randomised controlled study. Participants were either allocated to receive a tongue muscle training protocol (therapy group) or a sham protocol (control group) for 6 weeks. Adult patients previously diagnosed with moderate OSA (AHI via polysomnography between 15 and 30 events/h) who were registered in the database of the Sleep Investigation and Research Center of the Lausanne University Hospital and the Sleep Medicine Center of the Cliniques universitaires Saint‐Luc (Brussels, Belgium) were eligible if they presented a low adherence to CPAP therapy (mean use <4 h per night). Change in OSA severity classification between the diagnostic polysomnography and the AHI measured at inclusion was allowed. Exclusion criteria were craniofacial malformations, current use of hypnotic medications, history of stroke, a concurrent neuromuscular disease or a severe obstructive nasal disease. If participants were still on CPAP therapy, they were invited to discontinue this therapy at least 1 week before the start of the protocol.^29^ Eligible patients were identified in the database of both recruiting Centers and were invited to participate in this trial.

All included patients provided written informed consent to participate in the study. The study was approved by the local ethics committees (Commission Cantonale d'Ethique de la Recherche sur l'être humain [CER‐VD, Lausanne, central Ethic Committee] and the Comité d'Ethique Hospital‐Facultaire of Saint‐Luc‐UCLouvain [CEHF, Brussels, local Ethic Committee]) and is registered in ClinicalTrials.gov (NCT03846349).

### 2.2 Iowa oral performance instrument

Both measurements and training of the tongue were performed using the Iowa Oral Performance Instrument (IOPI, IOPI Medical LLC). The IOPI has demonstrated excellent interrater reliability to measure tongue strength and endurance^30^ and reference data have been published.^31^ The two models of the IOPI were used for the purpose of this study: the IOPI Pro (Model 3.1) and the IOPI Trainer (Model 3.2). The IOPI Pro device was used for the measurements. It consists of a portable device measuring the pressure that an individual can produce by squeezing a small air‐filled bulb with the tongue against the hard palate with an upward movement of the anterior part of the tongue. The pressure obtained is displayed on the LCD screen of the instrument and is expressed in kilopascals (kPa). A series of LED lights representing percentages in 10% increments of a manually set pressure allows to evaluate tongue endurance by assessing the time an individual can hold this set pressure. The IOPI Trainer device is similar to the IOPI Pro although it cannot measure pressure. It is intended for use in the home environment of the patient during the therapy period. A target pressure can be fixed and modified for the exercises protocol. The data of the training are stored in this device, allowing to measure compliance with the training protocol. A IOPI Trainer device was given to each participant randomised in the therapy group during the whole duration of their training programme.

### 2.3 Training protocol

The training protocol consisted in anterior tongue elevation strength and endurance tasks. Participants were instructed to perform the training exercises once a day, 4 times a week for 6 weeks. A typical session duration was 15 minutes. The load, sets and repetitions gradually increased each week to account for the expected strength and endurance improvement over the successive weeks and followed the recommendation of the American College of Sport Medicine.^32^

Tongue elevation strength task was performed by instructing participants to raise their tongue against the hard palate in order to squeeze the IOPI bulb positioned immediately posterior to the central incisors. In the first week of training, the participants had to perform 3 sets of 10 repetitions, while achieving 60% of their maximal elevation force (i.e. maximal pressure exerted on the tongue bulb) measured beforehand (see below). The rest between sets lasted 2 min. In the last week of training, they had to perform 4 sets of 12 repetitions while achieving 80% of their baseline strength value. The full training protocol is summarised in Table 1.

**TABLE 1:** Description of the training protocol

	Week 1	Week 2	Week 3	Week 4	Week 5	Week 6
*n* sessions/week	4	4	4	4	4	4
Strength task						
Load (% baseline Pmax)	60%	65%	70%	70%	75%	80%
*n* repetition/set	10	10	10	12	12	12
*n* sets/session	3	3	3	4	4	4
Endurance task						
Load (% baseline Pmax)	50%	50%	50%	50%	50%	50%
*n* sets/session	2	2	2	3	3	3

After the strength task, the participants were invited to complete a tongue isometric endurance elevation task. Participants were asked to maintain an isometric lingual pressure on the IOPI bulb equivalent to 50% of the baseline strength value until task failure, corresponding to a pressure drop >10% (visible by the LED lights of the IOPI device) for more than 2 s. During the first 3 weeks, participants had to perform 2 sets of this task interspersed with 2 min rest intervals. In the the last 3 weeks, the number of sets increased to 3 (Table 1).

The first session was realised face‐to‐face with the investigator. Oral and written instructions were given to patients to assist them in setting up the IOPI Trainer device independently. In addition, participants were contacted by phone once a week to ensure proper utilisation of the IOPI device.

### 2.4 Sham protocol

The sham protocol consisted in exhaling 10 repetitions for 3 consecutive sets with 2 min rest between series in a positive expiratory pressure device (Threshold PEP) with a resistance set at the lowest output (4 cmH_2_O), 4 sessions per week for 6 weeks. The resistance increased slightly from 4 to 6 cmH_2_O over the 6 weeks to simulate a real training programme and favour adherence of participants randomised in the control group.

### 2.5 Adherence

All patients had to fill a diary recording adherence to exercises (yes or no). Adequate adherence was defined by the completion of at least 75% of all exercise sessions. In the therapy group, all exercise sessions were recorded in the IOPI Trainer device, which was then additionally controlled.

### 2.6 Outcome measures

Physical characteristics of the subjects (body mass index, abdominal and neck circumference) and all the following outcome measures were evaluated pre‐ and post‐intervention.

#### 2.6.1 Polygraphy

Ambulatory respiratory polygraphy was performed at home using the Somnolter device (Nomics). The signals acquired were the following: chest and abdominal respiratory inductance plethysmography belts, nasal flow with a pressure transducer, oxygen saturation with a digital oximeter (Nonin; Nonin Medical) and mandibular movement (MM) recording. The MMs were recorded during polygraphy with a midsagittal MM magnetic sensor (Brizzy; Nomics), which measures the distance between two parallel, coupled, resonant circuits placed on the forehead and on the chin.^33^ The position and motion of the mandible is thus recorded during sleep. This signal has been shown to be a reliable marker of respiratory effort (RE) during sleep.^33^

Polygraphy scoring was performed by an experimented therapist (OC) blinded to the randomisation of the subjects and according to the guidelines provided by the American Academy of Sleep Medicine 2012.^34^ Accordingly, a minimum of 4 h of total sleep time was required for analysis.

The parameters recorded during ambulatory polygraphy were AHI, oxygen desaturation index, the minimal pulse oxygen saturation recorded during sleep (SpO_2_ min), the proportion of RE during the total sleep time and a respiratory effort index derived from the episodes of mandibular movement (MM‐REI).

#### 2.6.2 Tongue muscle assessment

The IOPI Pro device was used to assess tongue muscle elevation strength and endurance. Tongue strength is measured by obtaining maximal tongue elevation pressure. Anterior tongue elevation strength was assessed by asking the participants to squeeze as hard as possible and for 3 s the IOPI bulb positioned as mentioned above. Three trials interspersed by a 2 min resting period were allowed and the greatest value will be registered. Tongue endurance was assessed 5 min later by measuring the duration the participants were able to sustain the bulb squeeze at 50% of the maximal strength value recorded. Timing began when the participants achieved the target pressure and was discontinued when the recorded pressure dropped below 10% of the target for more than 2 s. The mean duration of two trials was reported. To ensure accurate measurement, calibration was checked and adjusted, if necessary, prior to obtaining measurements from each participant.

#### 2.6.3 Questionnaires

Subjective daytime sleepiness was measured by the Epworth Sleepiness Scale (ESS) which evaluate the propensity to sleep from ‘no chance of falling asleep’ (scored 0) to ‘high chance’ (scored 3) in eight different situations. Total score greater than 10 indicates excessive daytime sleepiness.^35^ Quality of sleep was evaluated with the Pittsburgh sleep quality index (PSQI), which is a questionnaire that evaluates seven sleep components on a scale from 0 (no difficulty) to 3 (severe difficulty). The results are expressed as a global score (ranging from 0 to 21). Total score greater than 5 indicates poor sleep quality.^36^ Finally, fatigue was assessed through the Pichot questionnaire.^37^ The questionnaire comprised 24 items including three homogenous sub‐scales of eight items each, measuring, respectively, the depressive mood, the asthenia‐fatigue and the anxiety dimensions. Only the asthenia‐fatigue scale was used in the protocol which consisted of eight questions scored from ‘0’ (not at all) to ‘4’ (extremely).

### 2.7 Randomisation

Patients were randomised in a 1:1 ratio arranged into block sizes of 6. Allocation concealment was performed using sequentially numbered sealed opaque envelopes prepared by an independent researcher not involved in the trial. The investigators (WP, NC, JT) opened the envelope after the collection of baseline measurements.

### 2.8 Data analysis

The primary outcome was the AHI recorded by polygraphy. Sample size was calculated to be 32 patients (16 per group) to have at least 80% power to detect a difference in mean AHI reduction of 5 units between both groups assuming a standard deviation of difference of 4.8 units and a two‐sided α level of .05^20^, ^38^ (PASS 14, NCSS, LLC). To anticipate a 10% risk of dropout rate, we aimed to recruit 36 patients.

Given the sample size, we chose to carry out non‐parametric tests. Therefore, data are presented as median and interquartile range (IQR). The results were analysed on a modified intention‐to‐treat basis, including all randomised participants who had at least started one session of the sham or training protocol. Wilcoxon test was applied for within‐group changes between the different time points. Between‐group differences were compared using Mann–Whitney *U* test. All tests were two‐sided and *p*‐values ≤.05 were considered significant. Statistical analyses were performed using SPSS version 27 (IBM).

## 3 RESULTS

### 3.1 Flow of participants through the study

**FIGURE 1:**
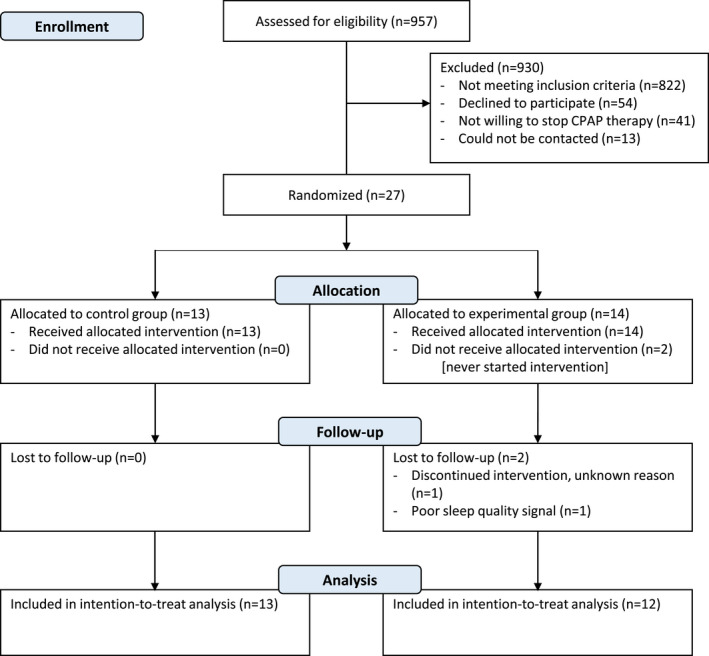
Study flow diagram

Between February 2019 and May 2021, 957 patients were screened from the sleep centers database. A total of 135 patients were eligible, and 27 (20%) were recruited (CONSORT flow diagram in Figure 1). Because of the slowing down of the recruitment process due to COVID‐19 pandemic, we could not reach the aimed sample size within the funding period. After excluding 2 patients who did never start the allocated intervention, 25 patients remained. Their baseline demographic data, sleep characteristics and symptoms are provided in Table 2. There were no differences in any characteristics between both groups at baseline. Despite all included patients had been diagnosed with moderate OSA as reported on medical records, 9 patients (36%) had an AHI < 15 events/h and 6 patients (24%) had an AHI > 30 events/h at baseline polygraphy assessment.

**TABLE 2:** Baseline characteristics of participants

Parameters	Control (*n* = 13)	Therapy (*n* = 12)	*p*‐value
	Median [IQR]	Median [IQR]	
Age (year)	56.0 [51.0–62.0]	48.0 [44.8–55.5]	.121
Sex, No. males (%)	6 (46)	8 (66)	.302
BMI (kg/m^2^)	28.9 [21.1–33.1]	26.5 [24.5–29.6]	.744
Neck circumference (cm)	37.0 [31.5–39.3]	38.0 [34.0–40.8]	.604
Abdominal circumference (cm)	102.0 [76.5–112.0]	93.5 [88.5–102.0]	.744
Total sleep time (min)	415.6 [383.1–472.4]	383.8 [348.1–412.2]	.157
Supine sleep time (%)	42.2 [31.7–62.0]	42.6 [21.4–60.3]	.644
AHI total (events/h)	16.8 [9.7–31.7]	18.9 [10.3–37.5]	.624
AHI Supine (events/h)	19.2 [13.4–36.1]	20.1 [16.7–43.6]	.514
AHI Nonsupine (events/h)	12.0 [8.1–22.3]	8.4 [5.6–23.9]	.765
ODI (events/h)	16.7 [8.1–31.6]	20.3 [9.4–37.7]	.786
MM‐REI (events/h)	25.2 [18.7–37.3]	30.7 [19.8–46.8]	.369
Respiratory effort (%)	47.2 [36.3–60.0]	47.0 [32.6–66.0]	.935
SpO_2_ min (%)	83.0 [78.0–87.0]	81.5 [80.0–86.8]	.935
Tongue Force (kPa)	62.0 [51.0–65.5]	54.5 [51.0–62.0]	.286
Tongue Endurance (s)	22.7 [18.8–29.3]	22.5 [17.0–31.4]	.807
ESS score	8.0 [4.5–11.0]	10.0 [4.5–15.3]	.445
PSQI score	7.0 [3.5–11.5]	8.0 [7.0–9.0]	.956
Pichot scale score	15.0 [8.5–19.5]	11.0 [2.3–21.0]	.604

*Note*: All data are presented are presented as median (25%–75%).

Abbreviations: AHI, apnoea‐hypopnea index; BMI, body mass index; ESS, Epworth Sleepiness Scale; MM‐REI: mandibular movement‐based respiratory effort index; ODI: oxygen desaturation index; PSQI, Pittsburgh sleep quality index questionnaire; SpO2 min, minimal pulse oxygen saturation value recorded during polygraphy.

### 3.2 Adherence to therapy

A total of 23 participants (92%) filled in and returned their training diary. One participant in the therapy group fulfilled only 46% of all exercise sessions. All other patients realised at least 75% of the training programme. Reported adherence with sham and study protocols was high in both groups (median [IQR] control: 100% [100–100]; therapy: 96% [79–100]). In the therapy group, there was no difference between the reported adherence on the diary and the adherence data recorded and retrieved from the IOPI device (mean difference: 0.03, 95% CI ‐2.29 to 2.36).

### 3.3 Effects of the tongue training protocol

**FIGURE 2:**
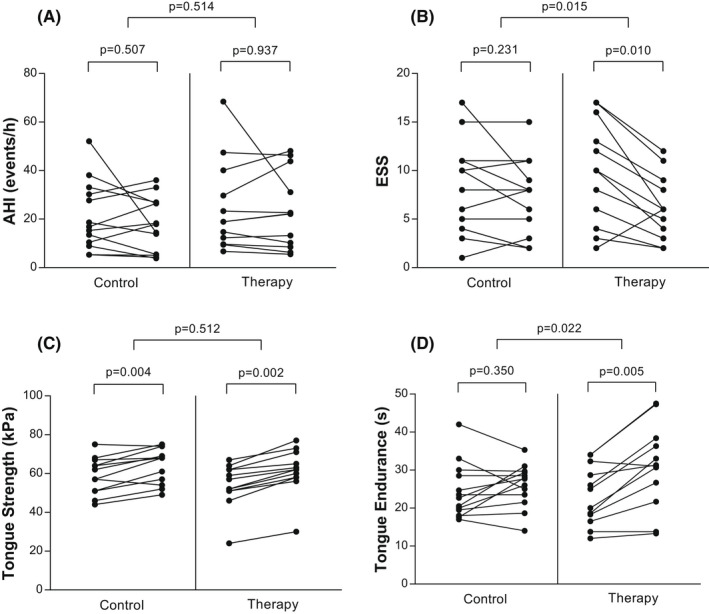
Effects of tongue training on OSA severity, daytime sleepiness and tongue muscle function. The graphs show individual pre‐ and post‐intervention values for (A) apnoea‐hypopnea index, (B) daytime sleepiness, (C) tongue elevation muscle strength and (D) tongue elevation isometric endurance in both control (sham intervention) and therapy (tongue training protocol) groups. AHI, apnoea‐hypopnea index, ESS, Epworth Sleepiness Scale

No changes in weight, neck and abdominal circumferences during the study period were observed in each group. In addition, there was no significant changes in AHI and other polygraphy‐derived parameters between groups at the end of the 6‐week trial period. In the control group, only tongue force significantly improved. In the therapy group, tongue force and endurance as well as subjective sleepiness, quality of sleep and fatigue significantly improved (Table 3, Figure 2). Comparing the changes between groups after 6 weeks of intervention, only the ESS score and tongue endurance differed significantly, in favour of the intervention group (Table 3, Figure 2). Per‐protocol analysis yielded to the same results.

**TABLE 3:** Anthropometric, symptom and sleep characteristics at baseline and after 6 weeks of randomisation

	Control (*n* = 13)			Therapy (*n* = 12)			Between‐group difference
Outcomes	Baseline	6 weeks	*p*‐value	Baseline	6 weeks	*p*‐value	*p*‐value
BMI (kg/m^2^)	28.9 [21.1–33.1]	28.9 [20.9–33.3]	.686	26.5 [24.5–29.6]	26.6 [24.5–29.6]	.144	.342
Neck circumference (cm)	37.0 [31.5–39.3]	37.0 [31.5–39.5]	.577	38.0 [34.0–40.8]	38.0 [34.0–41.0]	.301	.158
Abdominal circumference (cm)	102.0 [76.5–110.0]	103.0 [76.0–109.5]	.490	93.5 [88.5–102.0]	94.5 [89.5–103.0]	.414	.537
Total sleep time (min)	416 [383–472]	389 [299–469]	.382	384 [348–412]	358 [298–407]	.182	.999
Supine sleep time (%)	42.2 [31.7–62.0]	56.7 [38.4–67.2]	.807	42.6 [21.4–60.3]	51.2 [20.5–67.8]	.695	.957
AHI total (events/h)	16.8 [9.7–31.7]	17.9 [5.3–26.7]	.507	18.9 [10.3–37.5]	22.2 [8.9–40.6]	.937	.514
AHI Supine (events/h)	19.2 [13.4–36.1]	21.8 [17.2–30.0]	.861	20.1 [16.7–43.6]	28.0 [19.8–48.2]	.182	.353
AHI Nonsupine (events/h)	12.0 [8.1–22.3]	6.6 [3.8–13.6]	.152	8.4 [5.6–23.9]	6.6 [4.4–17.2]	.071	.723
ODI (events/h)	16.7 [10.3–31.6]	18.8 [5.0–26.2]	.650	20.3 [9.4–37.7]	22.4 [8.4–42.0]	.875	.624
MM‐REI (events/h)	25.2 [18.7–37.3]	29.0 [12.3–34.7]	.916	30.7 [19.8–46.8]	29.7 [22.5–46.4]	.754	.827
Respiratory effort (%)	47.2 [36.3–60.0]	41.9 [25.7–56.0]	.505	47.0 [32.6–66.0]	43.8 [33.4–65.9]	.875	.663
SpO_2_ min (%)	83.0 [78.0–87.0]	84.0 [80.0–86.5]	.167	81.5 [80.0–86.8]	83.5 [78.0–87.0]	.525	.563
Tongue Force (kPa)	62.0 [51.0–65.5]	68.0 [55.5–74.0]	**.004**	54.5 [51.0–62.0]	62.0 [58.0–69.5]	**.002**	.512
Tongue Endurance (s)	22.7 [18.8–29.3]	27.7 [22.5–29.5]	.350	22.5 [17.0–31.4]	31.2 [23.0–37.9]	**.005**	**.022**
ESS score	8.0 [4.5–11.0]	8.0 [4.0–10.0]	.231	10.0 [4.5–15.3]	6.0 [3.3–8.8]	**.010**	**.015**
PSQI score	7.0 [3.5–11.5]	6.0 [4.5–8.0]	.052	8.0 [7.0–9.0]	4.5 [3.3–6.0]	**.005**	.187
Pichot scale score	15.0 [8.5–19.5]	7.0 [6.5–23.0]	.674	11.0 [2.3–21.0]	6.5 [1.8–13.0]	**.047**	.286

*Note*: All data are presented are presented as median (25%–75%).

Abbreviations: AHI, aponea‐hypopnea index; BMI, body mass index; ESS, Epworth Sleepiness Scale; MM‐REI: mandibular movement‐based respiratory effort index; ODI: oxygen desaturation index; PSQI, Pittsburgh sleep quality index questionnaire; SpO2 min, minimal pulse oxygen saturation value recorded during polygraphy.

*p*‐value < .05 are bolded.

## 4 DISCUSSION

This study showed that a 6 weeks tongue elevation strength and endurance training programme did not influence OSA severity. However, improvements in tongue endurance and daytime sleepiness were observed.

The effects of the tongue task training protocol proposed is this study is not comparable to the acknowledged benefits of MFT on OSA severity. A recent review reported that AHI is reduced by approximately 50% in adults and 62% in children receiving MFT exercises.^16^ This therapy was also shown to reduce episodes of desaturation.^16^ However, the main drawback of MFT is adherence^22^ which has been reported to be around 80%, but it can be as low as 50%.^18^, ^39^ Key factors favouring adherence to prescribed exercises are, among other things, short duration of exercises, visual feedback, as well as exercise frequency monitoring.^40^, ^41^ These factors were integrated in our protocol by using the IOPI device and by constructing a non‐burdensome protocol, which may explain the high adherence level objectively measured in this study despite the realisation of exercises at home. O'Connor‐Reina et al. have recently found that 35% of their patients were nonadherent to an MFT protocol composed of exercises lasting 15 min/day and performed at a frequency of 5 days/week.^28^ It is worth noting that adherence to MFT programmes are related to improvements in AHI, OSA symptoms and tongue muscle strength.^28^, ^42^

Since we obtained good patient adherence with our training programme, the lack of change on AHI may be due to the fact that only the tongue was targeted in this study, whereas previous MFT studies proposed a combination of exercises covering various oropharyngeal structures. Therefore, we assume that focusing on tongue elevation tasks may not represent a sufficiently well‐rounded programme to elicit a detectable effect on polygraphy measurements. Interestingly, although we did not find a change in AHI or oxygen desaturation index, a large improvement in the ESS score was found in the tongue training group. In addition, 9 out of 12 participants (75%) had an ESS score decrease that exceeded three points, the minimum clinically important difference.^43^ Although this result should be interpreted with caution given that it is a secondary endpoint, it still argues in favour of a more efficient sleep. Likewise, albeit Randerath et al. did not find an improvement in AHI after a 8 weeks program of focused tongue muscle training by electrical intraoral neurostimulation, snoring episodes significantly decreased, suggesting a reduction in supraglottic resistance.^44^ Vranish and Bailey showed that 6 weeks of inspiratory muscle strength training reduced the number of arousals without achieving an improvement in AHI.^45^ It should be highlighted that respiratory events recorded by our ambulatory polygraphy device do neither measure the relative effort intensity of these events, nor the respiratory effort‐related arousals. Although these arousals generally do not wake the patient, this sleep fragmentation is considered as the primary cause of excessive daytime sleepiness in individuals with OSA.^46^ Therefore, a possible reduction in the intensity of respiratory efforts or in the number of arousals would not be captured.

Targeting tongue muscle exercises was based on anatomopathological data. Indeed, changes in the distribution of tongue muscle fibre types have been observed in untreated patients with OSA, making them more vulnerable to upper airway dilator muscles fatigue.^9^, ^13^ This may explain why some patients exhibit a poor genioglossus muscle responsiveness to negative pharyngeal pressure, a factor identified as contributing to the pathogenesis of OSA.^8^ The improvement in tongue endurance in the intervention group may thus have accounted for the improvement in OSA‐related symptoms. Future studies should determine if tongue endurance training is accompanied with a restoration of the proportion of fast‐ and slow‐twitch muscle fibres.

The human tongue comprises an extensive array of muscle fibres aligned at various angles, thereby allowing the tongue to generate an almost infinite variety of shapes and motions. Hydrostatic deformation of the human tongue is then the result of synergistic contractions of orthogonally aligned extrinsic and intrinsic fibres.^47^ We speculate that the integrative approach of the MFT interventions described in the studies of Diaferia et al.^12^ and Guimaraes et al.^20^ adequately addresses the upper airway dysfunction underpinning the physiopathology of OSA by stimulating synergistic co‐activation of extrinsic and intrinsic tongue fibres. However, it should be noted that a simple isometric tongue protrusion training during a 1 h session on seven consecutive days was associated with an increase in the tongue musculature corticomotor excitability and a significant decrease in AHI.^48^, ^49^ It remains unclear whether these changes are the result of a short but intensive training protocol, or the isometric tongue protrusion task, or a combination of both factors. Further investigations are thus needed to explore how specific tongue motion (protrusion, elevation, lateralisation) and function (endurance, force, power) may remodel upper airways and improve OSA severity.

Six‐week of tongue training protocol might also explain in part the lack of significant effect in the primary outcome. Indeed, published MFT protocols which have demonstrated clear benefits in patients with OSA typically lasted 2–3 months.^12^, ^20^, ^26^, ^50^ Ethical concerns influenced the choice of the 6 weeks training period. In fact, our patients were selected based on their low adherence to CPAP therapy, some having abandoned CPAP while others have been asked to discontinue CPAP during the protocol. A longer trial duration was deemed difficult to accept by clinicians and ethics committees. However, this duration was also based on a scientific rationale. As highlighted above, short tongue‐training protocols of 1 week have been found to reduce AHI and to induce neuroplastic changes in the corticomotor area of the tongue musculature.^48^, ^49^ In accordance with these findings, we observed an increase in the tongue muscle strength and endurance with our 6 weeks tongue training protocol. Whether or not this improvement in muscle function would be associated with a decrease in AHI was unknown and deserved to be studied.

The improvement of tongue muscle strength in the sham therapy was an unexpected finding. The sham therapy consisted of positive expiratory pressure at low resistance (4 to 6 cmH_2_O). Yanagisawa et al. found that expiratory muscle training can stimulate the genioglossus muscle, especially when the expiratory pressure load was high.^51^ This may explain the improvement in tongue muscle strength observed in the group who received sham therapy.

The main strength of our study is the objective measurement of patient adherence by the training device, which was missing in most previous trials. Our study had however several limitations. First, we did not achieve the estimated sample size due to the COVID‐19 pandemic. Nonetheless, the data collected in roughly three‐quarter of the anticipated sample size (achieved power: 70%) suggest that the recruitment of more patients would likely yield to the same conclusions. Second, selection bias could not be avoided. Inclusion of patients with moderate OSA was attempted based on a recent meta‐analysis that evaluated the impact of MFT.^16^ The vast majority of patients had moderate OSA, and the group data showed an average of 50% reduction in AHI. Based on these findings, we attempted to recruit this category of patients based on their initial diagnostic polysomnography to assess the effectiveness of an isolated tongue muscle elevation task protocol. However, some included patients had mild or severe OSA. The discrepancies between expected and measured baseline AHI could be due to different measurement methods (polysomnography at diagnosis vs polygraphy at start of the present study)^52^ or the natural course of the disease due to time elapsed between diagnosis and study inclusion. Nonetheless, recent studies have found that MFT also brings benefits in patients with mild or severe OSA.^14^, ^53^, ^54^ Therefore, albeit MFT protocols should not be confounded with our tongue task training protocol, we hypothesise that this selection bias had a negligible impact on our results. Third, we did not assess the phenotype traits of our cohort. Presumably, the best candidates for MFT are those who present low pharyngeal muscle responsiveness.^8^ Future studies aiming to identify individuals who will potentially benefit the most from this therapy are needed.^55^ Finally, we did not include anatomical variables in our exclusion criteria such as Mallampatti score, dental malocclusion and tongue tie. How these variables interact with the effects of tongue training in patients with OSA is unknown.

## 5 CONCLUSION

Our data suggest that 6 weeks of isolated tongue muscle elevation task has no effect on OSA severity.

## AUTHOR CONTRIBUTIONS

W. Poncin and O. Contal had full access to all of the data in the study and take responsibility for the integrity of the data and the accuracy of the data analysis. Concept and design: W. Poncin, O. Contal. Acquisition of data: W. Poncin, J. Tam, N. Correvon. Analysis or interpretation of data: All authors. Drafting of the manuscript: W. Poncin. Critical revision of the manuscript for important intellectual content: All authors. Statistical analysis: W. Poncin. Supervision: O. Contal.

## FUNDING INFORMATION

This was a researcher‐initiated study, supported with fundings from the HES‐SO University of Applied Sciences and Arts Western Switzerland, the European Respiratory Society, the Belgian Respiratory Society and the Foundation Saint‐Luc.

## CONFLICT OF INTEREST

All authors declared no conflict of interest.

### PEER REVIEW

The peer review history for this article is available at https://publons.com/publon/10.1111/joor.13369.

## Data Availability

The data that support the findings of this study are available from the corresponding author upon reasonable request.
